# Profiling Generalized Anxiety Disorder on Social Networks: Content and Behavior Analysis

**DOI:** 10.2196/53399

**Published:** 2025-03-20

**Authors:** Linah Alhazzaa, Vasa Curcin

**Affiliations:** 1 Department of Informatics King's College London London United Kingdom; 2 Department of Computer Science Princess Nourah bint Abdulrahman University Riyadh Saudi Arabia; 3 Department of Population Health Sciences King's College London London United Kingdom

**Keywords:** generalized anxiety disorder, mental health, Twitter, social media analysis, natural language processing

## Abstract

**Background:**

Despite a dramatic increase in the number of people with generalized anxiety disorder (GAD), a substantial number still do not seek help from health professionals, resulting in reduced quality of life. With the growth in popularity of social media platforms, individuals have become more willing to express their emotions through these channels. Therefore, social media data have become valuable for identifying mental health status.

**Objective:**

This study investigated the social media posts and behavioral patterns of people with GAD, focusing on language use, emotional expression, topics discussed, and engagement to identify digital markers of GAD, such as anxious patterns and behaviors. These insights could help reveal mental health indicators, aiding in digital intervention development.

**Methods:**

Data were first collected from Twitter (subsequently rebranded as X) for the GAD and control groups. Several preprocessing steps were performed. Three measurements were defined based on Linguistic Inquiry and Word Count for linguistic analysis. GuidedLDA was also used to identify the themes present in the tweets. Additionally, users’ behaviors were analyzed using Twitter metadata. Finally, we studied the correlation between the GuidedLDA-based themes and users’ behaviors.

**Results:**

The linguistic analysis indicated differences in cognitive style, personal needs, and emotional expressiveness between people with and without GAD. Regarding cognitive style, there were significant differences (*P*<.001) for all features, such as insight (Cohen *d*=1.13), causation (Cohen *d*=1.03), and discrepancy (Cohen *d*=1.16). Regarding personal needs, there were significant differences (*P*<.001) in most personal needs categories, such as curiosity (Cohen *d*=1.05) and communication (Cohen *d*=0.64). Regarding emotional expressiveness, there were significant differences (*P*<.001) for most features, including anxiety (Cohen *d*=0.62), anger (Cohen *d*=0.72), sadness (Cohen *d*=0.48), and swear words (Cohen *d*=2.61). Additionally, topic modeling identified 4 primary themes (ie, symptoms, relationships, life problems, and feelings). We found that all themes were significantly more prevalent for people with GAD than for those without GAD (*P*<.001), along with significant effect sizes (Cohen *d*>0.50; *P*<.001) for most themes. Moreover, studying users’ behaviors, including hashtag participation, volume, interaction pattern, social engagement, and reactive behaviors, revealed some digital markers of GAD, with most behavior-based features, such as the hashtag (Cohen *d*=0.49) and retweet (Cohen *d*=0.69) ratios, being statistically significant (*P*<.001). Furthermore, correlations between the GuidedLDA-based themes and users’ behaviors were also identified.

**Conclusions:**

Our findings revealed several digital markers of GAD on social media. These findings are significant and could contribute to developing an assessment tool that clinicians could use for the initial diagnosis of GAD or the detection of an early signal of worsening in people with GAD via social media posts. This tool could provide ongoing support and personalized coping strategies. However, one limitation of using social media for mental health assessment is the lack of a demographic representativeness analysis.

## Introduction

### Background

Mental health disorders are increasingly common, affecting approximately 1 billion people worldwide [1]. Among these mental health disorders, anxiety disorders are prevalent, with 3.94% of the world’s population affected in 2019 [2]. It is important to note that generalized anxiety disorder (GAD) is the most common anxiety disorder. According to the 2014 UK Adult Psychiatric Morbidity Survey, 5.9% of adults experienced GAD [3]. According to the *Diagnostic and Statistical Manual of Mental Disorders, Fifth Edition* (*DSM-5*) [4], GAD is characterized by extreme and persistent worry and anxiety about everyday activities lasting at least 6 months. Symptoms can be both mental and physical, including unrealistic anxiety, inability to relax, restlessness, headaches, rapid heart rate, and difficulty breathing [4].

Untreated GAD, in the long term, may lead to physical and mental health complications [5]. It also reduces the quality of life by affecting work productivity and physical and social functions [6]. In addition, GAD burdens health care systems and the economy. It increases health care costs due to higher use of services and decreases work productivity, leading to more days of disability [7]. Despite the availability of effective GAD treatments, only 43.2% of those who have it receive treatment [8]. Consequently, early detection of GAD could significantly reduce all these burdens and improve quality of life.

People use technology in nearly every aspect of their lives. Social media has become more popular, reaching 4.8 billion users worldwide in 2023 [9]. Individuals often share their mental health status on social media as a way of reducing isolation, seeking support, overcoming stigma, increasing awareness, coping with challenges, and sharing experiences without the feeling of being unfairly judged [10]. This generates valuable data on users’ posts, profiles, and web-based activities that can provide insights into their mental health conditions. Therefore, exploiting data extracted from social networks is essential to revealing users’ mental health statuses.

Consequently, this study presented an approach to identifying GAD markers via social media. By analyzing both linguistic and behavioral patterns on Twitter (subsequently rebranded as X), this approach constructed a comprehensive digital profile of individuals with GAD, distinguishing them from the control group. The methodology integrated analytical tools, such as Linguistic Inquiry and Word Count (LIWC; Pennebaker Conglomerates, Inc) for detailed linguistic analysis and GuidedLDA for thematic exploration, yielding a multidimensional understanding of language and topic patterns unique to GAD. Moreover, a thorough behavioral analysis examined hashtag use frequency, follower counts, interaction patterns, and other aspects, identifying GAD-associated behaviors. This integrated approach highlights social media’s potential as a mental health assessment resource and support by identifying 47 statistically validated digital markers of GAD.

In summary, our primary contributions are (1) characterizing the digital markers of GAD by analyzing social media content and behaviors of those who have it, by identifying distinct patterns of language use, thematic topics, and behavioral traits that differentiated them from individuals in the control group; (2) conducting an extensive content-based analysis that leveraged LIWC for a detailed linguistic-based analysis and GuidedLDA for a comprehensive thematic analysis; (3) identifying significant behavioral differences based on user and tweet-level features, such as hashtag use frequency and interaction patterns with other users, as well as measuring the correlation between the themes derived from GuidedLDA and various metadata attributes for both the GAD and control groups; and (4) presenting our findings on 47 distinct GAD-associated digital markers through comprehensive statistical analysis.

### Related Works

The widespread use of social media networks and the availability of massive data inspire researchers to investigate the characteristics of mental illnesses through user-generated content and users’ activities on social media platforms. Defining these characteristics helps detect mental illnesses via social media, which could help psychiatrists assess a patient’s diagnosis and recovery progress. Many studies have used natural language processing (NLP) and machine learning techniques for mental illness prediction via social networks. Several studies have focused mainly on depression prediction via social networks [11-24], and some have examined anxiety disorders [25-30], posttraumatic stress disorder [31-33], and eating disorders [15,34-37]. Therefore, identifying digital markers of mental health conditions could improve detection accuracy.

Textually generated content on social media platforms has been studied to identify indicators of mental health problems. Several studies in psycholinguistics have explored the link between language use and psychological health. Most of these works have used LIWC, a text analysis program that uses predefined dictionaries to capture various dimensions, such as social, emotional, and psychological states. Many studies have analyzed word use to extract psycholinguistic lexical features for identifying depression [11,12,18,25,33,38-40], posttraumatic stress disorder [31,33,40], bipolar disorder [25,33,40], eating disorders [25,34,35], seasonal affective disorder [25,33,40], anxiety disorders [25,29], obsessive-compulsive disorder [25], and schizophrenia [25,41].

A few works have studied the word use associated with psychological health in different languages to identify mental illnesses. The study by Hao et al [28] used a Chinese version of LIWC to investigate psychological characteristics from posts collected from Sina Weibo, a popular Chinese social media platform. In the study by Peng et al [42], they extracted emotional features for depression based on the Chinese Affective Lexicon Ontology, a Chinese emotional dictionary, by adding the depression class to other emotional classes in the emotional dictionary and excluding the surprise class because it is unrelated to depression.

Several studies have analyzed textual content using topic modeling techniques to reveal hidden topics in social media posts and define their relevance to mental health disorders. Latent Dirichlet allocation (LDA) is a widely used topic modeling technique in previous research, including studies on depression [11,18], anxiety disorders [29,30], anorexia [34], schizophrenia [41], and a set of mental health disorders [26].

Because of the significance of leveraging social interaction metadata from users with mental health conditions, some studies have been conducted to understand how individuals with a low mental health status interact on social media platforms. Therefore, studying users’ behaviors could provide insights into their mental health characteristics. A survey [43] investigated the correlation between web behaviors and psychological health, concluding that some web activities, such as internet addiction, instant messaging, and web-based gaming, are highly related to mental health disorders.

In the context of social media, previous research has examined social media users’ activities as an indicator of their mental health status. Vedula and Parthasarathy [19] studied the behaviors of people with depression on Twitter, whereas Peng et al [42] analyzed the depressive characteristics of Sina Weibo users based on posts and user profiles. In the study by de Choudhury et al [44], they investigated changes in new mothers’ characteristics related to mood and behavior in the period following childbirth based on some measures of linguistic style, emotional state, and ego network (ie, the number of followers and followees, as well as engagement with the Twitter community). The work by Wang et al [35] characterized individuals with eating disorders via Twitter based on their social interactions, behaviors, and psycholinguistic properties.

It is worth noting that using social media data for mental health assessment offers valuable insights. However, it also presents ethical considerations. Privacy is the key ethical concern when using publicly available data [45,46]. Therefore, to maintain users’ privacy, it is essential to apply anonymization techniques to the datasets collected from social media. This process involves removing all information that could reveal a person’s identity.

Although previous research has conducted content analysis of users with anxiety disorders via social media, a limited number of works have focused mainly on GAD. Therefore, a thorough study of GAD characteristics on social media platforms is needed. Accordingly, this study provided an in-depth and detailed profiling of users with GAD via social media. We conducted a content analysis, including linguistic and thematic analysis, the web-based behavior of users with GAD, and the correlation between themes and users’ behaviors. These analyses could contribute to a better understanding of and provide insights into GAD markers, helping extract significant features used in GAD detection via social media.

The rest of this paper is organized as follows. The methodology is outlined in the next section. The results of our work are then presented. The last section highlights the findings and discusses the limitations and possible future directions to extend our research.

## Methods

### Overview

To identify GAD characteristics in social media users, we performed several steps. The first step was collecting data using the Twitter application programming interface (API) [47] to obtain posts from both GAD and control groups. Second, a set of data-cleaning procedures was applied. Third, we investigated the dataset based on different aspects, including linguistic analysis, thematic analysis, and user behavior analysis.

**Figure 1:**
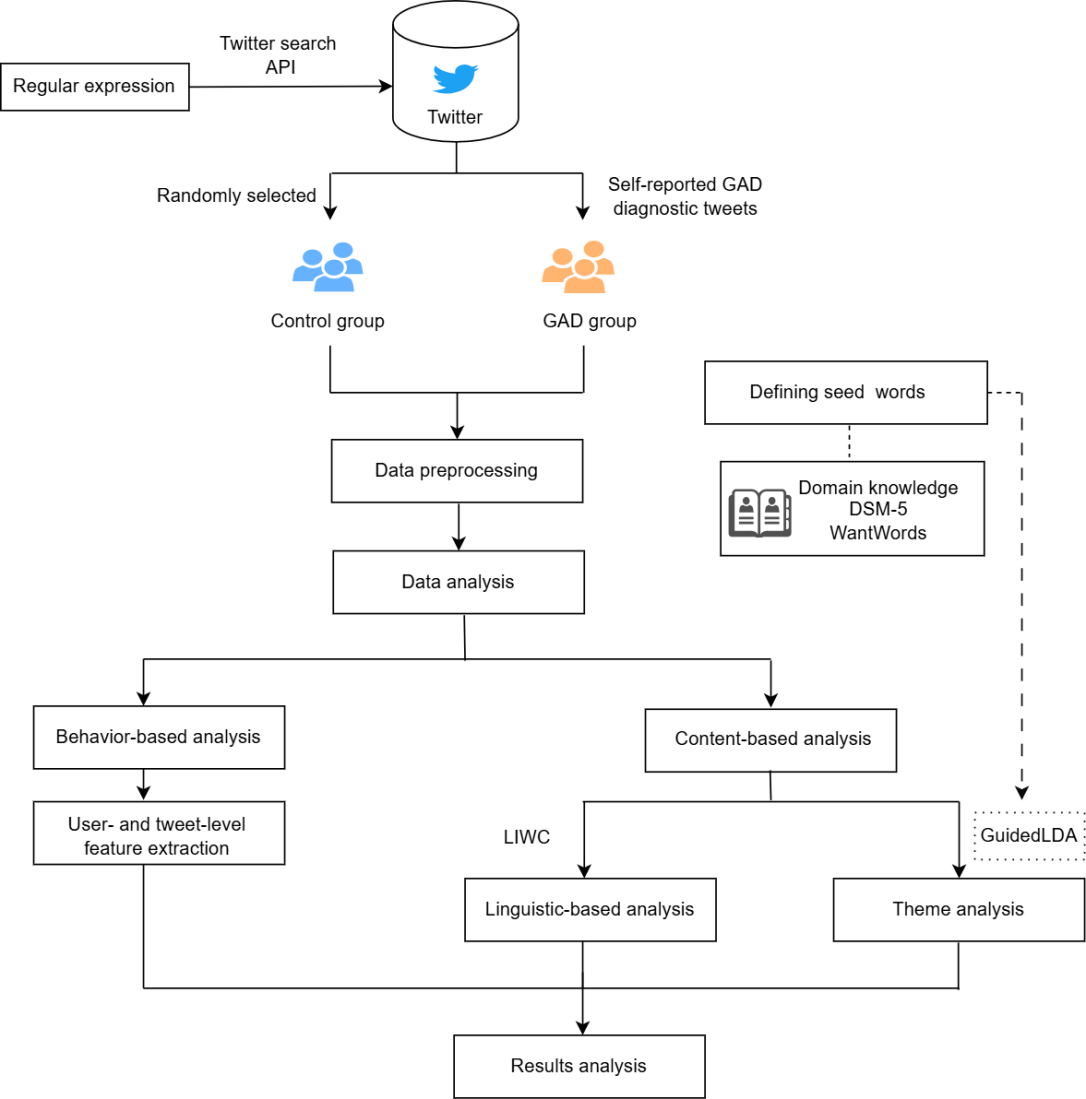
The analytical workflow of generalized anxiety disorder (GAD) profiling via social media involved collecting data from Twitter for the GAD and control groups; performing a set of data preprocessing steps; and conducting a thorough analysis, including behavior-based and content-based analysis. API: application programming interface; DSM-5: Diagnostic and Statistical Manual of Mental Disorders, Fifth Edition; LIWC: Linguistic Inquiry and Word Count.

LIWC was used for the linguistic-based analysis, whereas the thematic analysis was based on the GuidedLDA algorithm. The user- and tweet-level metadata were used to analyze user behaviors, and several features were extracted from tweets and user profiles. We then explored the results to shed light on GAD markers derived from the content and behavior analysis. Figure 1 illustrates the analytical workflow of GAD profiling via social media. The subsequent sections provide an in-depth explanation of each step.

### Data Collection

The data were collected from Twitter, a widely used social media platform. The reason for choosing Twitter was its popularity as a social media platform, with approximately 369 million active accounts every month worldwide in 2022 [48]. Another reason was that Twitter offers a significant amount of data for researchers on health topics because of the enormous number of users, its data accessibility, and the lack of difficulty retrieving and searching data [49].

The Twitter platform provides an API for pooling public posts, searching for relevant ones using regular expressions, and streaming real-time tweets. Through the Twitter API, we collected a dataset encompassing users with GAD and users comprising the control population. Identifying individuals with GAD was done by following the method proposed by Coppersmith et al [40]. This method uses a regular expression to search for tweets that include the diagnosis statement for a condition, such as “I was diagnosed with [condition name],” using the Twitter API. In our study, a group of users with GAD was selected using a regular expression to search tweets that contained the following statement—“I was diagnosed with Generalized Anxiety Disorder (GAD)”—to ensure that a user had publicly stated that they had been diagnosed with GAD. This resulted in 3445 tweets from 2721 unique potential users in the GAD group.

The Twitter API returned tweets comprising all words that matched the given expression, requiring a manual verification procedure to eliminate tweets unrelated to the GAD diagnosis statement. This process ensured that the collected tweets specifically pertained to a self-reported diagnosis of GAD of users who posted them, so a manual review was conducted to ensure that the tweets did not mention quotations or stories of someone else with GAD. As a result, of the 2721 unique potential users, 1500 (55.13%) users with GAD were included in the dataset.

**Figure 2:**
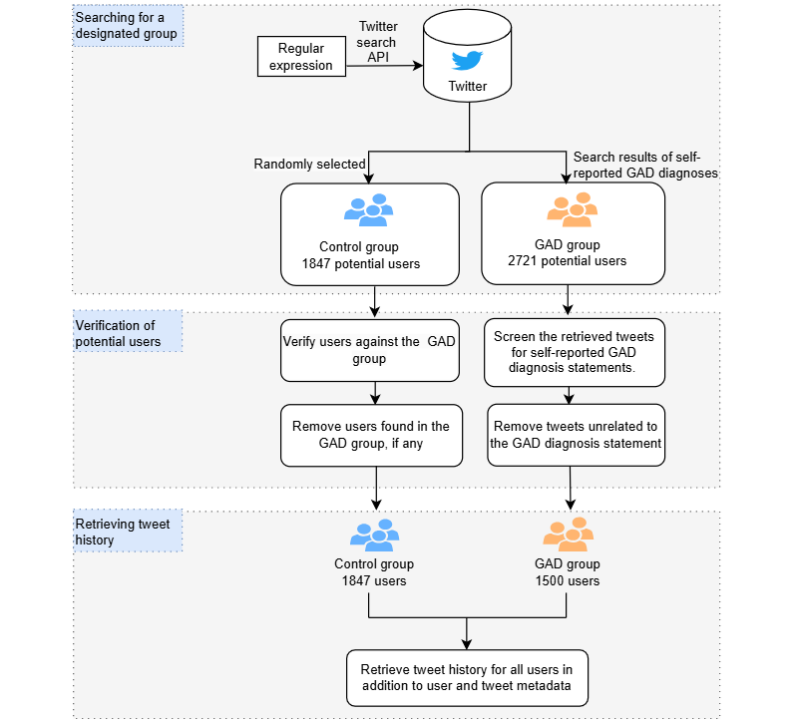
The data collection process from Twitter for the generalized anxiety disorder (GAD) and control groups. It encompassed searching for a target group, verifying potential users, and retrieving their tweets. API: application programming interface.

The control group consisted of randomly selected users representing a general population sample. This resulted in 1847 control group users being included in the dataset. In instances in which users were found to be present in both the GAD and control groups, they were excluded from the control group. The most recent tweets for users with GAD and control users were then retrieved considering the limitation of retrieving a maximum of 3200 tweets per user imposed by the Twitter API. Figure 2 illustrates the process of collecting the dataset.

As shown in Tables 1 and 2, the user and tweet metadata were also retrieved for each profile to study the users’ behaviors and social engagement. It is worth noting that this study only collected data from users whose accounts were public, and all the collected data were deidentified to protect privacy.

**Table 1:** A description of each type of user-level metadata [50] that was retrieved from Twitter for each user in both the generalized anxiety disorder and control groups.

Feature	Description
User ID	A unique ID for a particular user
Status count	It shows how many tweets were posted by a user.
Description	The user biography briefly describes the user’s account.
Verified	It shows whether the user’s account is verified.
Account creation date	It shows the date and time of the account’s creation.
Follower count	The total amount of users who follow an account
Friend count	The number of users that are currently being followed by an account
Listed count	The number of public lists that a user has joined
Favorite count	The total number of tweets that have been marked as favorites

**Table 2:** A description of tweet-level metadata [51] that were retrieved from Twitter for each user in both the generalized anxiety disorder and control groups.

Feature	Description
Tweet ID	A unique ID for a tweet
Full tweet	The tweet text
Tweet created at	The date and time when a tweet was posted
Favorite count	The number of likes for a tweet
Retweet count	The number of retweets for a tweet
Reply to user ID	If a tweet is a response to another user’s tweet, this attribute will show that user’s unique identifier.

### Data Preprocessing

#### Overview

Several preprocessing steps were undertaken to prepare the 2 collections of raw tweets for further analysis. Data transformation was applied first. We transformed the raw data (tweets) into a structured format in which the columns were the user ID, full tweet, and favorite count, among other data, as described in Tables 1 and 2. Therefore, each row included 1 tweet for a user ID. These tweets were then further processed as outlined in the following sections.

#### Filtering Out

**Figure 3:**
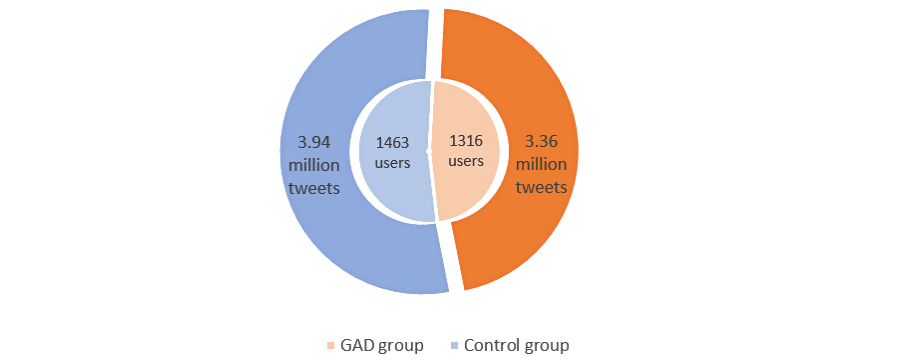
The statistics for the collected data are presented for the generalized anxiety disorder (GAD) and control groups showing the number of users and tweets for both groups.

As this study focused on analyzing posts in English, the dataset was filtered to exclude users with <80% of tweets in English. We then removed non–English-language tweets. Users with <100 tweets were excluded from the dataset. This resulted in a final dataset with a total of 7,313,769 tweets posted by 2779 users. Of these 2779 users, 1316 (47.36%) had been diagnosed with GAD and had posted a total of 46.04% (3,367,581/7,313,769) of the tweets, whereas 1463 (52.64%) of the users constituted the control group with a total of 53.96% (3,946,188/7,313,769) of the tweets. The average number of tweets was 2558.95 (SD 1315.98) for each individual with GAD, whereas it was 2697.33 (SD 918.30) for each user in the control group. Figure 3 illustrates the statistics of collected data for the GAD and control groups, showing the number of users and tweets for each group.

HTML tags and URLs were also eliminated. As part of the preprocessing step, tweets were converted into lower case to prevent case sensitivity in the subsequent data-cleaning steps. Furthermore, identifiable usernames or posts were eliminated to protect users’ privacy.

The following data preprocessing steps were standard and have been applied in previous NLP-related studies [52,53].

#### Tokenization

Tokenization is applied to split tweets into a list of tokens (ie, words) using the Natural Language Toolkit (NLTK; Team NLTK) tokenizer. The output of this process becomes the main input for punctuation and stop word removal, word normalization, and part-of-speech (POS) tagger steps.

#### Punctuation and Stop Word Removal

Stop words, such as articles and prepositions, are widely used in any natural language, but they are commonly ignored in text analysis because of their low meaning value and high frequency. Thus, we removed them from the data using the standard stop word list provided in the NLTK. Punctuation was also removed as it adds noise to the dataset. This step was conducted using regular expressions and the NLTK library.

#### POS Tagger

The POS tagger was applied to assign each token the appropriate POS, such as noun, verb, adverb, and adjective. This process enhances the quality of preprocessing. The POS must be specified for every token using the NLTK POS tagger to obtain the correct lemma.

#### Lemmatization

As words have different forms, it is essential to unify the word representation to analyze the text. Applying word normalization techniques eliminates the different forms of the same word. Lemmatization is a technique that reduces words to their base form (lemma) using a corpus, such as WordNet. We applied lemmatization to reduce each token to its root using the NLTK lemmatizer based on the WordNet dictionary (The Trustees of Princeton University).

### LIWC for Text Analysis

#### Overview

Several studies have revealed a correlation between language use and an individual’s mental health status. Therefore, we investigated the differences in word use patterns between users with GAD and control users to identify underlying potential indications of GAD. We used LIWC [54], a text analysis tool equipped with >100 built-in dictionaries to reveal individuals’ psychological, social, and behavioral patterns from text, each including words, word stems, and emoticons [54]. These words could be classified into one or multiple categories or dictionaries.

The process of text analysis using LIWC involves several steps. These steps are reading the dataset, comparing every word with each dictionary entry, and calculating the percentage of words related to each built-in dictionary. A quantitative summary of all LIWC dimensions is then generated, and the user-level means for all dimensions are found. Finally, these results could be analyzed to capture underlying psychosocial indicators.

As mental health impacts our way of thinking, behaving, and feeling [1], we proposed 3 measurements based on LIWC dictionaries. These measurements, namely, cognitive style, personal needs, and emotional expressiveness, aimed to study the differences in language use between users with GAD and control users. We carefully defined these measures inspired by the method in the studies by Pavalanathan and de Choudhury [55] and Ernala et al [56]. This method is based on leveraging insights from an extensive review and synthesis of previous research in psychology and psycholinguistics [57-59]. The potential implications of studying linguistic style are significant as it could provide a deeper understanding of the linguistic markers of GAD and potentially lead to more effective interventions.

#### Cognitive Style

The cognitive style quantifies how individuals think, talk about their thinking, or retrieve information. It is based on the cognitive dimension of LIWC to measure related categories, including insight, causation, discrepancy, tentativeness, certitude, and differentiation. In addition, the memory category is used to help measure the retrieval or forgetfulness of information. The results of these analyses could reveal a decline in cognition associated with poor mental health status in an individual with GAD, including memory recall issues, difficulty reasoning, and decision-making inability.

#### Personal Needs

Identifying personal needs helps maintain mental well-being, which could be helpful to separate the GAD group from the control group as the former may not sufficiently satisfy various personal needs. Therefore, we investigated some personal needs, including essential needs and self-esteem, based on LIWC dictionaries. The essential needs involve being physically and mentally healthy and feeling safe, including seeking support and maintaining a healthy life, which was assessed using some LIWC categories (ie, *food*, *substances*, *death*, *want*, *need*, and *lack*).

Self-esteem needs involve belief in personal value and ability, which also impacts how to communicate with others. Accordingly, measuring self-esteem needs includes several categories (ie, *reward*, *curiosity*, *risk*, *interpersonal conflict*, and *communication*). The results of these categories could reflect mental well-being based on the definition of mental well-being according to the World Health Organization, which is a person’s ability to recognize their skills, deal with everyday life stressors, effectively produce and accomplish, and participate in the community [60].

#### Emotional Expressiveness

Emotional expressiveness measurement assesses a person’s tendency to express various emotions through language use. We used the *negative tone*, *anxiety*, *anger*, *sadness*, and *swear words* categories to measure emotional expressiveness, which could identify the patterns of expressing emotional sentiments that are common among people with GAD.

### GuidedLDA

Topic modeling is a content analysis approach for uncovering hidden themes in textual data. One common topic modeling technique is LDA, an unsupervised machine learning mechanism. LDA helps extract topics with no advanced knowledge of themes. However, implementing LDA on a large dataset may generate meaningless topics [61]. To leverage previous research and the domain knowledge of GAD in topic modeling, we adopted the GuidedLDA technique for extracting the common topics discussed among people with GAD.

GuidedLDA is a semisupervised LDA algorithm based on setting predefined seed words to extract hidden themes of a particular direction and interest. Unlike the LDA topics initialized using randomly selected words, each seed word has a high probability of belonging to its particular topic in the initialization stage of GuidedLDA. We defined some possible seed words for each candidate topic related to GAD by relying on domain knowledge and previous research on GAD. We explain the configuration of GuidedLDA in the *Thematic Analysis* section.

### Twitter Metadata

In addition to retrieving the user-level and tweet-level metadata shown in Tables 1 and 2, we extracted several features derived from Twitter metadata. These included tweet and behavior-based features, as shown in Table 3.

**Table 3:** A set of extracted features derived from Twitter metadata.

Feature	Description
FF^a^ ratio	The ratio of followers to followees per user
Number of mentions	The average number of mentions per tweet
Number of tweets	The number of tweets per user across their history
Number of replies	The number of replies per user across their history
Reply-to-tweet ratio	The ratio of replies to the total number of tweets per user
Number of hashtags	The number of participating hashtags per user across their history
Hashtag ratio	The ratio of tweets including hashtags to the total number of tweets per user
Number of retweets	The number of retweets per user across their history
Retweet-to-tweet ratio	The ratio of retweets to the total number of tweets per user
Total number of mentions per user	The number of mentions per user across their history
Total number of mentions without replies per user	The number of mentions per user without considering mentions associated automatically with replies across their history
Mention ratio	The ratio of tweets with other people mentioned to the total number of tweets per user
Total tweets with favorites	The total number of tweets that received at least one favorite per user
Tweets with favorites ratio	The ratio of total tweets that received at least one favorite to the total tweets per user
Total tweets with retweets	Total tweets that received at least one retweet per user
Tweets with retweets ratio	The ratio of total tweets that received at least one retweet to the total tweets per user
Tweets per day	The average number of tweets per day per user
Replies per day	The average number of replies per day per user
Retweet per day	The average number of retweets per day per user
Mentions per day	The average number of tweets including mentions per day per user
Hashtag engagement per day	The average number of tweets including hashtags per day per user

^a^FF: follower-to-followee.

### Data Analysis

We aimed to compare various features between individuals with GAD and control individuals. Therefore, the analysis involved several statistical methods, including the 1-tailed, 2-sample *t* test; *P* value for statistical significance; and Cohen *d* for effect size. First, descriptive statistics were calculated for each content-based and behavioral-based feature for both groups, including means and SDs to summarize the data.

Next, we conducted the 1-tailed ,2-sample *t* test to compare the means of the 2 groups. This test evaluated whether the mean differences between these groups were statistically significant, with a *P* value of ≤.05 indicating statistical significance. The Cohen *d* was also computed to measure the effect size to provide insights into the practice effect. The possible magnitude of these differences ranged from small to large. According to Cohen [62] and Sawilowsky [63], the effect sizes are interpreted as small if Cohen *d*<0.20, as medium if the Cohen *d* is between 0.20 and 0.50, and as large if the Cohen *d* is between 0.50 and 0.80. The *P* value and Cohen *d* provide insights into the results’ statistical significance and practical importance.

### Ethical Considerations

This study used publicly available data without direct user interaction or intervention, so an ethics review was unnecessary. All usernames, IDs, and tweets were de-identified to ensure privacy. In addition, data that could disclose personal identifiers were carefully omitted to maintain user privacy and anonymity.

## Results

This section presents the results of exploring the data in terms of linguistic, thematic, and behavior analyses.

### Linguistic-Based Analysis

#### Overview

We found differences in language use across different aspects, including cognitive style, personal needs, and emotional expressiveness, between the individuals in the GAD group and those in the control group. Table 4 provides the summary statistics of these differences based on the LIWC categories for people with GAD compared to those without GAD. Consequently, these differences in the LIWC categories demonstrate a correlation between language use and the mental health status of people with GAD.

**Table 4:** A comparison of linguistic-based measurements between the generalized anxiety disorder (GAD) and control groups based on the Linguistic Inquiry and Word Count categories^a^.

Category		GAD group, mean (SD)	Control group, mean (SD)	*P* value	Cohen *d*
**Cognitive style (percentage of total word count)**					
	Insight	1.59 (0.57)	0.90 (0.65)	<.001	1.13
	Causation	1.028 (0.39)	0.60 (0.44)	<.001	1.03
	Discrepancy	1.39 (0.47)	0.78 (0.58)	<.001	1.16
	Tentativeness	1.40 (0.48)	0.74 (0.56)	<.001	1.27
	Certitude	0.57 (0.29)	0.30 (0.31)	<.001	0.90
	Differentiation	2.15 (0.71)	1.17 (0.88)	<.001	1.23
	Memory	0.10 (0.07)	0.07 (0.09)	<.001	0.37
**Personal needs (percentage of total word count)**					
	Need	0.35 (0.18)	0.20 (0.18)	<.001	0.83
	Want	0.38 (0.25)	0.23 (0.21)	<.001	0.65
	Lack	0.11 (0.20)	0.06 (0.07)	<.001	0.33
	Reward	0.11 (0.22)	0.15 (0.46)	.003	0.11
	Risk	0.19 (0.12)	0.11 (0.12)	<.001	0.67
	Curiosity	5.79 (1.31)	3.82 (2.30)	<.001	1.05
	Substances	0.05 (0.17)	0.03 (0.06)	<.001	0.16
	Food	0.48 (0.33)	0.32 (0.44)	<.001	0.41
	Death	0.19 (0.19)	0.11 (0.13)	<.001	0.49
	Interpersonal conflict	0.27 (0.17)	0.18 (0.19)	<.001	0.50
	Communication	3.50 (1.64)	4.80 (2.35)	<.001	0.64
**Emotional expressiveness (percentage of total word count)**					
	Negative tone	4.39 (1.36)	4.24 (1.73)	.01	0.10
	Anxiety	0.18 (0.26)	0.06 (0.08)	<.001	0.62
	Anger	0.17 (0.12)	0.09 (0.10)	<.001	0.72
	Sadness	0.22 (0.22)	0.13 (0.15)	<.001	0.48
	Swear words	0.77 (0.74)	0.47 (0.72)	<.001	2.61

^a^*P* values were calculated using a 1-tailed, 2-sample *t* test, with *P*≤.05 indicating statistical significance, and the effect size is reported using the Cohen *d*.

#### Differences in Cognitive Style

The GAD group had higher values for all categories used for assessing cognitive style than individuals in the control group, showing statistically significant differences between the 2 groups (*P*<.001) for insight (Cohen *d*=1.13), causation (Cohen *d*=1.03), discrepancy (Cohen *d*=1.16), tentativeness (Cohen *d*=1.27), certitude (Cohen *d*=0.90), differentiation (Cohen *d*=1.23), and memory (Cohen *d*=0.37), as shown in Table 4. Tweets posted by users with GAD included more certainty, discrepancy, and differentiation words, almost 2 times more than tweets posted by users in the control group. In addition, their tweets contained nearly twice as many tentative words as the control group. The results also indicated that the GAD group used nearly 1.5 times more memory words related to recollecting and forgetting, such as *forget* and *remember*, than the control group.

#### Differences in Personal Needs

Regarding essential needs, the results indicated that the tweets of the GAD group contained approximately 1.8 times more words from the *want*, *need*, and *lack* categories than the tweets of the control group. Moreover, the GAD group showed increased use of *substances*, *death*, and *food* words. The mean values of these categories were 0.05, 0.19, and 0.48, respectively, for the GAD group, which were higher than those for the control group.

Regarding self-esteem needs, the number of reward words in the control group was higher than that for the GAD group. Another result was a lower number of communication words in the GAD group than in the control group. In contrast, 1.5 times more words related to interpersonal conflict were observed in the GAD group compared to the control group.

Table 4 highlights the significant differences in most categories of personal needs, underscoring the depth of these differences. This was evident in the *need* (Cohen *d*=0.83), *want* (Cohen *d*=0.65), *lack* (Cohen *d*=0.33), *risk* (Cohen *d*=0.67), *curiosity* (Cohen *d*=1.05), *food* (Cohen *d*=0.41), *death* (Cohen *d*=0.49), *interpersonal conflict* (Cohen *d*=0.50), and *communication* (Cohen *d*=0.64) categories. Although the *reward* and *substances* categories were statistically significant (*P*=.003 and *P*<.001, respectively), the effect size was small (Cohen *d*=0.11 and 0.16, respectively).

#### Differences in Emotional Expressiveness

The GAD group expressed more negative tones. Notably, their tweets also showed extensive use of anxiety-related words, 3 times more than the control group. In addition, their tweets expressed anger and sadness more frequently. Moreover, the GAD group used a higher number of swear words.

We found statistical significance (*P*<.001) as well as a significant effect size for most features, including anxiety (Cohen *d*=0.62), anger (Cohen *d*=0.72), sadness (Cohen *d*=0.48), and swear words (Cohen *d*=2.61). However, the effect size of the negative tone was small (Cohen *d*=0.10).

### Thematic Analysis

#### Overview

We present the results of the thematic analysis, which included 4 primary themes: symptoms, life problems, relationships, and feelings. The symptoms theme was derived from major symptoms according to the *DSM-5* for GAD. Other themes were derived from previous research [64] highlighting how GAD may affect an individual’s perceived life satisfaction in different aspects such as relationships, health, and work. The seed words of the symptoms theme were defined based on GAD symptoms and the health-related problems of GAD according to the *DSM-5*. To generate seed words for other themes, we used a reverse dictionary named WantWords (The Natural Language Processing Group, Tsinghua University) [65] to obtain words that related semantically to the descriptions of these themes. Table 5 shows a sample of seed words for each theme.

**Table 5:** A sample of seed words used by GuidedLDA for each theme.

Theme	Seed words
Symptoms	“Worry,” “concern,” “anxiety,” “twitch,” “treatment,” “restlessness,” “concentration,” “muscle,” “mental,” and “health”
Life problems	“Business,” “money,” “work,” “school,” “job,” “salary,” “promotion,” “college,” “office,” and “experience”
Relationships	“Wife,” “husband,” “parents,” “kids,” “friends,” “partners,” “spouse,” “fiancee,” “fiance,” and “father”
Feelings	“Feel,” “cry,” “respect,” “release,” “support,” “emotion,” “disclosure,” “care,” “sadness,” “sense,” and “sorry”

#### Symptoms Theme

The symptoms theme reflects the GAD symptoms and other health problems related to GAD among those who have it that were expressed in their tweets. Some tweets showed how individuals with GAD have extreme anxiety about various things, and others represented how individuals with GAD tend to expect the worst scenario for any issue. In addition, users with GAD often posted about experiencing difficulty concentrating and overthinking plans. Other tweets showed that people with GAD tend to experience tiredness and sleep disturbance because of insomnia.

The results also demonstrate that many people with GAD discuss their health status, therapy, and treatment with others. Some users also discussed how excessive worry caused other health problems, such as heart conditions and digestive issues.

#### Life Problems Theme

This theme denotes how individuals with GAD worry about various issues, including financial status, work, and school. Some users with GAD tended to share with others how GAD negatively impacted work because of difficulty concentrating on any task and accomplishing it on time, reducing work performance. Some users with GAD discussed the difficulty maintaining employment and the increased fear of financial strain. Other tweets showed that several people with GAD discussed the impact of GAD on their academic performance.

#### Relationships Theme

This theme highlights that people with GAD have impairments in relationships with partners, family members, and friends. Many users tended to discuss their problems in relationships with others. We found that some tweets showed that individuals with GAD had fewer relationships for several reasons, such as having trouble expressing their feelings, having poor self-esteem, and being irritable with others. In addition, some users discussed how GAD significantly affected their marriage, whereas some individuals reported that GAD caused the weakening of their relationships with their partners and decreased enjoyment of activities with them.

#### Feelings Theme

This theme indicates that several users with GAD tend to express and share their feelings and emotions with others on social media. We found that they disclosed their feelings to obtain support and help from professionals or other users or raise awareness of mental health issues.

#### Summary of Thematic Analysis Results

**Figure 4:**
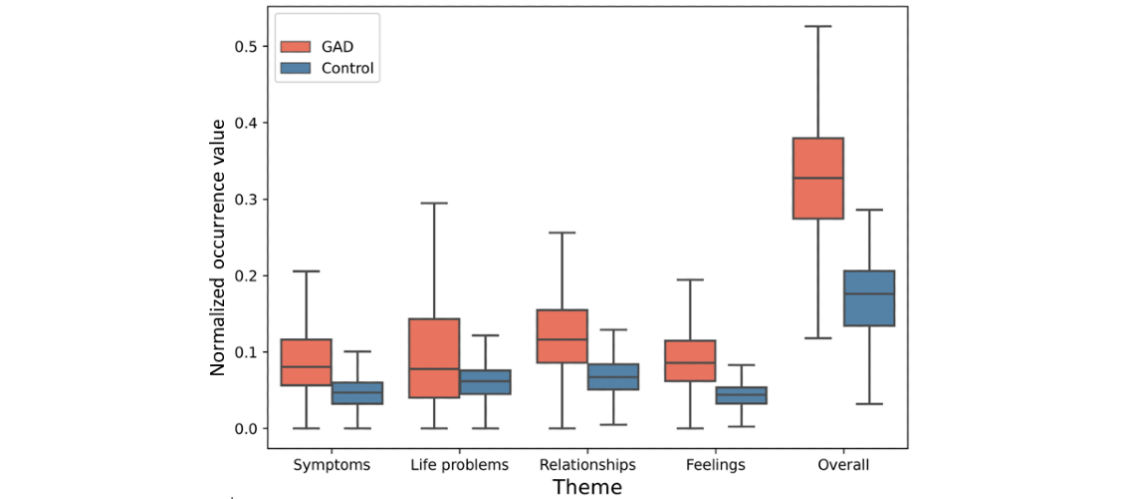
The frequency of the GuidedLDA-based themes per tweet for each user profile in both groups. GAD: generalized anxiety disorder.

In Figure 4, the GuidedLDA-based thematic analysis shows that users in the GAD group had a higher frequency of these themes per tweet than those in the control group for every theme. The results indicate that the GAD group tended to discuss life problems and other themes 1.5 times and approximately 2 times more than the control group, respectively. Table 6 shows the statistically significant differences for all themes between users with GAD and control users. We found statistically significant differences (*P*<.001) and significant effect sizes (Cohen *d*>0.50) for most themes. Examples of paraphrased tweets for each theme can be found in Multimedia Appendix 1.

**Table 6:** A comparison of themes between the generalized anxiety disorder (GAD) and control groups^a^.

Theme	GAD group, mean (SD)	Control group, mean (SD)	*P* value	Cohen *d*
Symptoms (percentage of total tweets per user profile)	10 (8)	5 (2)	<.001	0.86
Life problems (percentage of total tweets per user profile)	11 (10)	7 (7)	<.001	0.46
Relationships (percentage of total tweets per user profile)	13 (8)	7 (3)	<.001	0.99
Feelings (percentage of total tweets per user profile)	9 (5)	4 (2)	<.001	1.31

^a^*P* values were calculated using a 1-tailed, 2-sample *t* test, with *P*≤.05 indicating statistical significance, and the effect size is reported using the Cohen *d.*

### Behavioral Analysis

In this section, an analysis is presented to study the patterns of individuals with GAD concerning their behaviors and activities on Twitter. Moreover, we measured the correlation between the GuidedLDA-based themes and users’ behaviors, including hashtag participation, volume, interaction patterns, social engagement, and reactive behavior.

#### Participation in Hashtags

Participating in hashtags shows the tendency of users to discuss various topics in public with others. The analysis showed that people with GAD participated in fewer hashtags than people without GAD. In addition, the results showed different preferences for the types of hashtag topics followed by people with GAD compared to people without GAD. People with GAD preferred to participate in hashtags related to their mental health status (eg, *#anxiety* and *#mentalhealth*), therapy, and treatments (eg, *#ketaminetherapy* and *#ketwell*). In addition, they liked to follow hashtags where they had the opportunity to discuss their mental health with others to seek support and reduce isolation (eg, *#MentalHealthMatters*).

Many people with GAD tended to participate slightly more in hashtags related to current events regarding health, politics, and other matters (eg, *#news* and *#COVID19*) than those without GAD. On the other hand, people without GAD followed hashtags related to daily life, ranging from family to entertainment, more than individuals with GAD (eg, *#NBATwitter* and *#movie*). We found that people with GAD tended to participate less using hashtags that those without GAD were usually more active with. The mean value of users’ tweets per day containing a hashtag in the GAD group was 0.2 (SD 0.54; median 0.06, IQR 0-0.42), whereas the mean value of users’ tweets per day containing a hashtag for those without GAD was significantly higher at 0.53 (SD 1.13; median 0.13, IQR 0-0.89; *P*<.001).

We also measured the ratio of all tweets that participated in a hashtag to the overall tweets. We found that 15% (591,928/3,946,188) of tweets from the control group included hashtags compared to 8.6% (289,611/3,367,581) of tweets from the GAD group. The median value for the former was 9% (IQR 6.75%-13.5%), whereas the median value for the latter was 4.7% (IQR 3.53%-7.06%).

**Figure 5:**
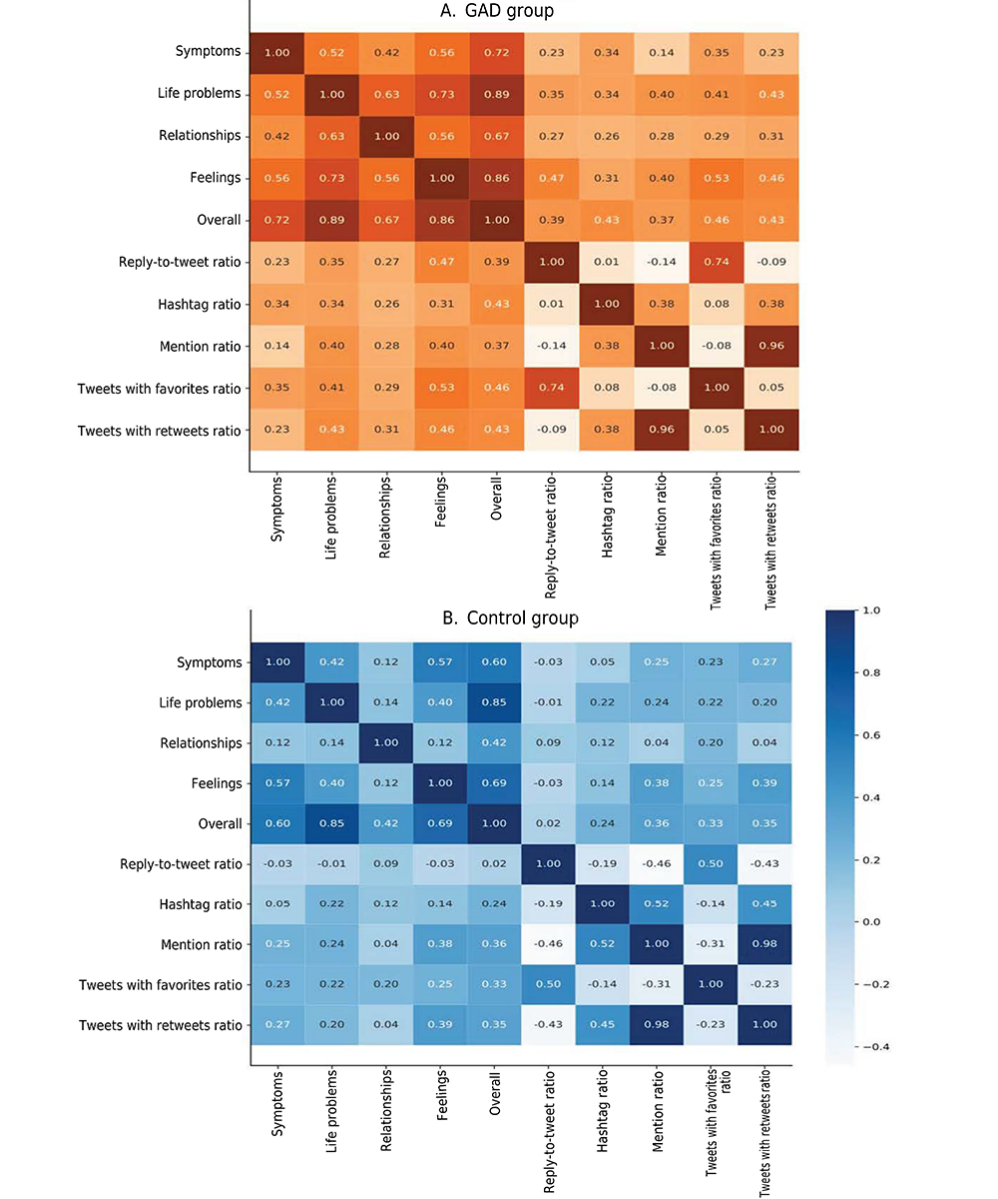
The correlation between the GuidedLDA-based theme frequency and metadata for (A) the GAD group and (B) the control group in our dataset. GAD: generalized anxiety disorder.

However, those in the GAD group who participated in hashtags mainly focused on sharing their symptoms, as shown in Figure 5. We also identified a higher correlation (ρ) between all 4 identified themes overall and hashtag ratio than for those in the control group. This indicates that individuals without GAD participated in hashtags that were less correlated with these themes. The highest value for ρ was observed when individuals with GAD shared their symptoms with others (0.34 compared to 0.05 for the control group).

#### Metadata of Engagement Analysis

We explored the 2 groups through metadata analysis, such as how they interacted with each other in terms of replies, mentions, number of followers and followees, and volume of tweets.

##### Volume

**Figure 6:**
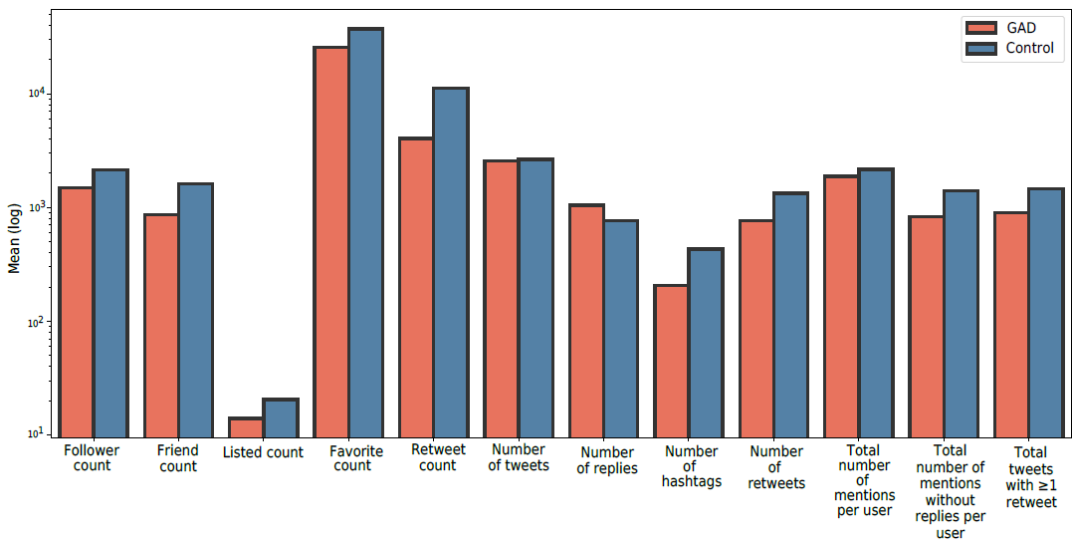
A comparison of mean values across different volume metrics between the generalized anxiety disorder (GAD) and control groups.

We measured the volume of several aspects for each user from their profile history. Our scope spanned 12 aspects to be compared between the GAD and control groups, as shown in Figure 6. In general, we found that individuals in the control group had 46.93% and 30.5% more friends and followers, respectively, and had a 31.53% higher listed count. In addition, we found that the control group was 52.33% more active in the participation in hashtags than the GAD group and tended to have 64.11% and 40.85% more retweets and mentions, respectively.

In addition, the control group tended to retweet popular tweets 42.47% more than the GAD group. However, we noticed a 36.01% lower volume of replies for the control group than for people with GAD. This might be due to the fact that the GAD group tends to be more reactive than proactive, which will be discussed later in the *Reactive Behavior* section.

Table 7 shows the statistical significance of the volume features. We found statistical significance and significant effect sizes for the number of replies (Cohen *d*=0.33), number of hashtags (Cohen *d*=0.51), number of retweets (Cohen *d*=0.62), total number of mentions per user (Cohen *d*=0.30), total number of mentions without replies per user (Cohen *d*=0.63), and total tweets with retweets (Cohen *d*=0.60). There were no statistically significant differences in the follower count and listed count features (Cohen *d*=0.06 and 0.07, respectively).

The results also showed a statically significant difference in friend count, favorite count, retweet count, and number of tweets. However, the effect size was small for these features (Cohen *d*=0.14, 0.20, 0.14, and 0.08, respectively).

**Table 7:** Comparing the volume metrics between the generalized anxiety disorder (GAD) and control groups^a^.

Feature	GAD group, mean (SD)	Control group, mean (SD)	*P* value	Cohen *d*
Follower count	1492.90 (7433.32)	2148.25 (12,479.92)	.09	0.06
Friend count	859.56 (2016.48)	1619.79 (7567.63)	<.001	0.14
Listed count	14.03 (65.89)	20.56 (111.17)	.06	0.07
Favorite count	25,480.23 (40,851.50)	37,215.05 (70,582.59)	<.001	0.20
Retweet count	4033.26 (25,873.79)	11,239.02 (70,861.73)	<.001	0.14
Number of tweets	2559.78 (1315.98)	2650.30 (918.30)	.04	0.08
Number of replies	1042.95 (873.12)	766.84 (792.72)	<.001	0.33
Number of hashtags	205.19 (305.56)	430.50 (539.18)	<.001	0.51
Number of retweets	767.65 (773.00)	1334.42 (1029.41)	<.001	0.62
Total number of mentions per user	1871.37 (1082.88)	2167.46 (909.44)	<.001	0.30
Total number of mentions without replies per user	828.42 (779.71)	1400.62 (1019.23)	<.001	0.63
Total tweets with retweets	902.59 (797.51)	1453.47 (1012.96)	<.001	0.60

^a^*P* values were calculated using a 1-tailed, 2-sample *t* test, with *P*≤.05 indicating statistical significance, and the effect size is reported using the Cohen *d.*

##### Interaction Patterns

The mention and reply features on Twitter could reflect the interaction among Twitter users. Therefore, we analyzed the ratio of mentions and replies for people with and without GAD to study the extent of interaction in these 2 groups. We found that individuals with GAD had a lower mention ratio than those without GAD, indicating less interaction with others.

Another indication of a user interacting with the community is acting positively and communicating more with others. On Twitter, users tend to mention someone within their tweets to ask for participation in their opinion. Another example is to retweet others’ ideas for either endorsement or to pass on an interesting tweet to their audience for further discussion. We found that control users tended to include more mentions per day than people with GAD. The mean number of mentions per day for the control group was 1.57 (SD 2.31; median 0.69, IQR 0.30-4.95). This is, in fact, more than double that of people with GAD (mean 0.78, SD 1.2; median 0.39, IQR 0.25-2.60).

For more confirmation of such behavior, we also found that 51% (2,012,555/3,946,188) of tweets from the control group had a mention, whereas 31.5% (1,060,788/3,367,581) of tweets included a mention in the GAD group. The mentions comprised those receiving a reply and those solely mentioned as both are technically considered *mentions* in the Twitter community. In Figure 5, users in the GAD group show a high correlation with mentions when talking about their life problems and relationships (approximately 0.4 and 0.28 compared to 0.24 and 0.04 for the control group, respectively).

However, the symptoms theme showed a lower correlation with mentions for the GAD group than for the control group (0.14 compared to 0.25). We found that the control group tended to talk directly to someone about health issues. In contrast, the GAD group associated health discussions with a hashtag to receive more attention and used direct mentions to seek support for life problems and relationships.

In addition, the retweets per day and their ratio to the overall tweets may indicate endorsement of others’ tweets or the desire to pass them on to their audience for further discussion. We found a mean of 1.63 (SD 2.31; median 0.74, IQR 0.50-5.19) retweets per day for the control group and 0.86 (SD 1.26; median 0.44, IQR 0.40-2.87) retweets per day for the GAD group. Moreover, 48% (1,894,170/3,946,188) of the control group’s tweets were retweets, whereas 28.91% (973,567/3,367,581) of the GAD group’s tweets were retweets.

However, we also observe in Figure 5 that, in the GAD group, there is a high correlation between both the feelings and life problems themes and higher likes and retweets received from their audience when they tweet (ρ of 0.53 and 0.41 in the GAD group compared to 0.25 and 0.22 in the control group, respectively). This result might suggest that the GAD group interacts with others when they share the effects of GAD on their life, seeking support and a way to release and express their emotions.

##### Social Engagement

We used the number of followers and followees to measure the social engagement among users. The results showed that people with GAD had fewer followers and followees than those without GAD, as shown in Figure 6. This might reflect that the GAD group was less social than the control group and tended to limit their relationships.

**Figure 7:**
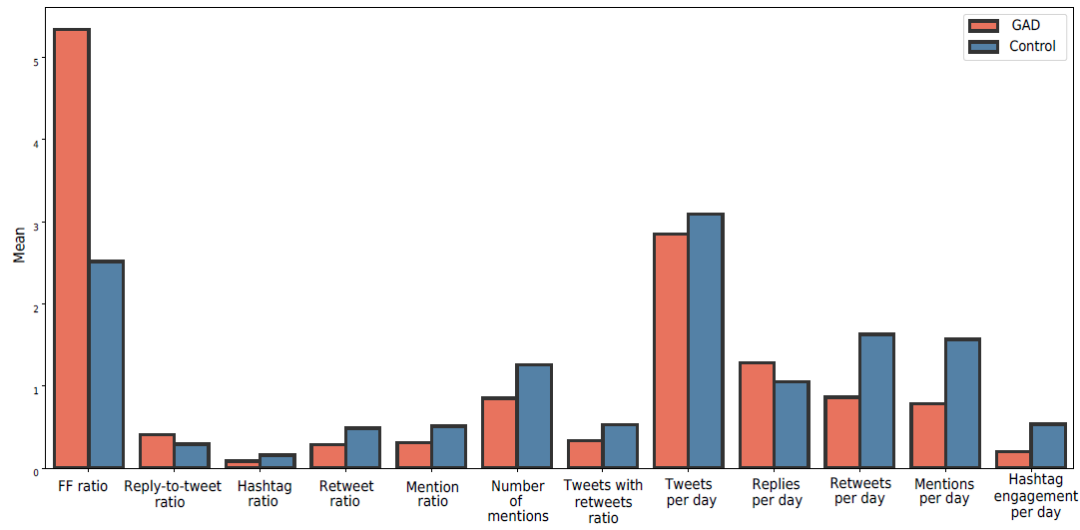
A comparison of mean values across different features between the generalized anxiety disorder (GAD) and control groups. FF: follower-to-followee.

However, we found that users with a large follower-to-followee ratio were associated with GAD, as shown in Figure 7. This result could be due to these users being more well known, making them more likely to receive criticism from individuals who are unfamiliar with them. In addition, people with GAD had fewer friends than the control group. To summarize these cases, we identified 2 categories within the GAD group. The first category was those with high pressure from a large number of followers, such as celebrities, whereas the second category was those who tended to live alone and have fewer friends.

**Figure 8:**
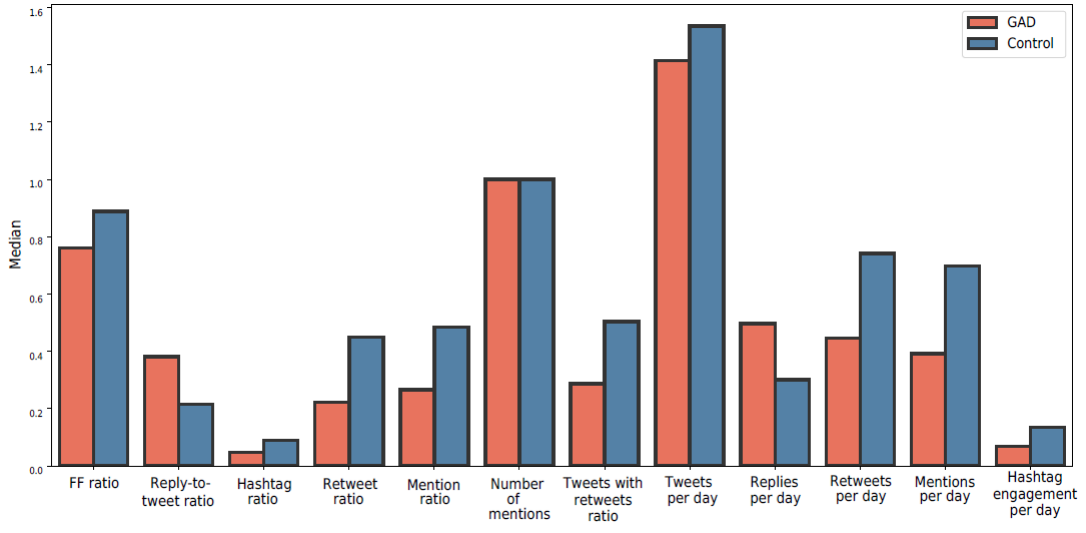
A comparison of median values across different features between the generalized anxiety disorder (GAD) and control groups. FF: follower-to-followee.

The mean value of the follower-to-followee ratio in the GAD group was 5.34 (SD 93.82). On the other hand, the control group had a mean follower-to-followee ratio of 2.51 (SD 15.53). The large value for the SD is due to those who had a high number of followers and experienced pressure from their audience, leading to some GAD symptoms. Therefore, we measured the median for both groups, with 0.76 (IQR 0.25-157.91) and 0.88 (IQR 0.25-26.89) for the GAD and control group, respectively. Figure 7 shows the first category within the GAD group, whereas Figure 8 shows the second category.

##### Reactive Behavior

We identified that people with GAD tended to reply more than write new tweets compared to those in the control group. Figure 7 shows that the mean value of users’ replies per day for the GAD group was 1.28 (SD 2.12), whereas the mean value of users’ replies per day in the control group was slightly lower at 1.05 (SD 2.05). The margin was even larger in terms of median values at 0.49 (IQR 0.35-4.40) and 0.30 (IQR 0.25-4.08) for the GAD and control groups, respectively. This may indicate a common aspect of those in the GAD group being more reactive to others rather than engaging proactively.

To clarify this, we also measured the ratio of replies to total tweets, which points to the same behavior. The mean value of this ratio for the GAD group was 0.41 (SD 0.24), and the median was 0.38 (IQR 0.30-0.82). In the control group, the mean value of the ratio of replies to total tweets was 0.29 (SD 0.26), and the median was 0.21 (IQR 0.18-0.69). We confirm the reactive behavior in Figure 5, where there is a higher correlation (ρ) for the GAD group between the *symptoms* theme, *life problems* theme, *relationships* theme, *feelings* theme, and the 4 identified themes overall and the reply-to-retweet ratio at 0.23, 0.35, 0.27, 0.47, and 0.39 compared to the control group at –0.03, –0.01, 0.09, –0.03, and 0.02, respectively. Table 8 shows the statistical significance for most of the behavioral features.

We found statistical significance and significant effect sizes for the reply-to-tweet ratio (Cohen *d*=0.47), hashtag ratio (Cohen *d*=0.49), retweet ratio (Cohen *d*=0.69), mention ratio (Cohen *d*=0.68), tweets with retweets ratio (Cohen *d*=0.68), retweets per day (Cohen *d*=0.41), mentions per day (Cohen *d*=0.42), and hashtag engagement per day (Cohen *d*=0.38).

There were no statistically significant differences in the follower-to-followee ratio and tweets per day features (Cohen *d*=0.04 and 0.07, respectively). The results also show a statistically significant difference in mention count and replies per day. However, the effect size was small for these features (Cohen *d*=0.17 and 0.11, respectively).

**Table 8:** Comparing the behavior-based metrics between the generalized anxiety disorder (GAD) and control groups^a^.

Feature	GAD group, mean (SD)	Control group, mean (SD)	*P* value	Cohen *d*
FF^b^ ratio	5.34 (93.82)	2.52 (15.53)	.28	0.04
Reply-to-tweet ratio	0.41 (0.24)	0.29 (0.27)	<.001	0.47
Hashtag ratio	0.087 (0.11)	0.16 (0.18)	<.001	0.49
Retweet ratio	0.29 (0.24)	0.49 (0.33)	<.001	0.69
Mention ratio	0.32 (0.23)	0.51 (0.32)	<.001	0.68
Number of mentions	0.85 (0.84)	1.26 (3.27)	<.001	0.17
Tweets with retweets ratio	0.34 (0.23)	0.53 (0.32)	<.001	0.68
Tweets per day	2.85 (3.69)	3.09 (3.56)	.82	0.07
Replies per day	1.29 (2.12)	1.06 (2.05)	.004	0.11
Retweets per day	0.87 (1.27)	1.63 (2.32)	<.001	0.41
Mentions per day	0.79 (1.20)	1.57 (2.31)	<.001	0.42
Hashtag engagement per day	0.20 (0.54)	0.54 (1.14)	<.001	0.38

^a^*P* values were calculated using a 1-tailed 2-sample *t* test, with *P*≤.05 indicating statistical significance, and the effect size is reported using the Cohen *d*.

^b^FF: follower-to-followee.

## Discussion

### Principal Findings

This study characterized the GAD digital markers using social media content and behavior analysis of individuals with GAD. The results show the importance of studying psychological states reflected in language. Investigating the word use patterns in people with GAD revealed differences in all the defined measurements (ie, cognitive style, personal needs, and emotional expressiveness) compared to individuals without GAD. First, all measurements of cognitive style were higher for those with GAD, including certainty, discrepancy, and tentativeness, which may reflect several GAD markers. One possible explanation for why users with GAD expressed more certainty is that they have difficulty dealing with uncertainty, which is aligned with the work by Miranda and Mennin [66]. Another possible reason is that people with GAD tend to have a negative outlook toward future events because of persistent anxiety [66]. The high number of tentative and discrepancy words in their tweets might be attributed to having difficulty putting worries aside or letting them go, having difficulty making decisions, and being indecisive, or it might also indicate inability to focus, consistent with the work by Koerner and Dugas [67].

In addition, individuals with GAD tended to use more memory words, which might reflect memory problems, including recalling and forgetfulness. This result might reveal some GAD symptoms (ie, difficulty concentrating and excessive worry) that may contribute to emerging memory issues, which is aligned with the work by Butters et al [68]. Second, we also found differences in personal needs regarding essential and self-esteem needs. All measurements of essential needs were higher for those with GAD. The increased use of word categories (ie, *want*, *need*, and *lack*) might indicate the desire to seek support and help or raise mental health awareness, which agrees with the work by Fine et al [69].

We also observed a higher number of references to food and death in the GAD group. This might indicate that GAD is more likely to be associated with unhealthy lifestyle behaviors, which is aligned with the work by Moselhy et al [70]. A possible reason for the increased use of food words in the GAD group might be the relationship between GAD and increased food intake, which agrees with the studies by Moselhy et al [70] and Fonseca et al [71]. Regarding self-esteem needs, people with GAD used fewer words related to rewards but more words related to curiosity and risk. This result might be explained by the fact that users who experience GAD have difficulty accomplishing tasks because of excessive anxiety, inability to focus, and overthinking, which is consistent with the work by Habib and Laidey [72].

Moreover, increased use of interpersonal conflict words and decreased use of communication words were observed in the GAD group. This might be due to being irritable and nervous, which are symptoms of GAD. Finally, the results also indicate differences in emotional expressiveness between the 2 groups. The increased negative tones, anger, anxiety, and sadness in tweets posted by individuals with GAD might be due to excessive anxiety, which is aligned with the work by Mennin et al [73], which found difficulties in emotion regulation among people with GAD.

Our findings highlight the importance of thematic analysis in quantifying GAD signals. We found that users with GAD tended to post more about their symptoms, health status, life issues, friendship problems, and feelings. Interestingly, there were significant differences in all themes—symptoms, life problems, relationships, and feelings—between the GAD group and the control group. We note that relying on the *DSM-5* and previous work [64] related to the impact of GAD on individual life enhanced our analysis to generate themes more related to GAD, resulting in a detailed thematic analysis to distinguish GAD signals.

We found that the dominant themes in tweets posted by users with GAD tended to focus on life problems and relationships, followed by feelings and symptoms. This result may be explained by the fact that worsening symptoms and difficulty with emotion regulation contribute to difficulty handling life and relationship issues.

The other key finding was that GAD signals can be found in users’ digital activities. The behavioral analysis focused on users’ activities based on Twitter metadata regarding hashtag participation, volume, interaction patterns, social engagement, and reactive behaviors. We found that the GAD group used fewer hashtags than those without GAD, meaning that people with GAD might have fewer interactions with the public. These findings might indicate that people with GAD tend to be isolated by having fewer relationships and limiting their interaction with people, which is consistent with the work by Henning et al [64] and Revicki et al [74] that shows that GAD is associated with heightened social functioning impairment.

We also observed that people with GAD have different preferences regarding topics for participating in hashtags compared to users without GAD. We found that people with GAD preferred to participate more in hashtags related to mental health disorders and treatments. This might be due to their low mood or desire to seek support, raise mental health awareness, or overcome mental health stigma. The results also showed that users with GAD were more likely to follow hashtags about the latest news, which might reflect their low mood and contribute to heightened anxiety levels, which is aligned with the work by Cheun et al [75].

In addition, we found that users without GAD tended to have more followees, mentions, retweets, and hashtags. These findings might suggest that people with GAD are less active and tend to limit their interaction with others. This might reflect that people with GAD have impairments in relationships, which is in agreement with the work by Henning et al [64].

Moreover, this analysis measured the correlation between the GuidedLDA-based themes and users’ behaviors. The results showed a high correlation between hashtag ratio and overall themes for the GAD group compared to the control group. For users with GAD, the results identified a high correlation between the mention ratio and the life problems and relationships themes, whereas the symptoms theme had a lower correlation with the mention ratio. This might indicate that the GAD group tends to discuss their health issues using related hashtags to obtain more attention and use mentions to seek direct help for life problems and relationship issues.

Our findings regarding language use patterns are consistent with the research presented in the studies by Coppersmith et al [25] and Shen and Rudzicz [29], which identify linguistic patterns for individuals with GAD. Regarding topic analysis, our study supports the conclusions of both the studies by Shen and Rudzicz [29] and Gkotsis et al [30] that individuals with GAD tend to focus on specific topics in their discussions. In our study, we comprehensively extended previous work by characterizing linguistic and thematic markers associated with GAD and providing a more detailed and in-depth examination of the textual content of individuals with GAD. Regarding the behavior analysis, previous studies [19,35,42,44] have covered the behaviors of individuals with a low mental health status on social media. Our exploration comprehensively analyzed how people with GAD interact and express themselves on the web. It highlights unique behavioral patterns that may contribute significantly to a deeper understanding of the disorder in the digital context. To the best of our knowledge, this is the first study that comprehensively explores the behaviors on social media of people with GAD, along with investigating the correlation between thematic content and users’ behaviors on social media platforms. Therefore, our work aims to expand our understanding of the unique digital markers of GAD related to linguistic, thematic, and behavioral aspects.

It is worth noting that using publicly available social media data for mental health research raises ethical issues, including concerns about users’ privacy and informed consent [45,46]. To address these ethical concerns, a comprehensive strategy to anonymize the collected datasets is essential to prevent the identification of users. This involves removing all identifiable information, such as usernames and real names, and paraphrasing tweets to ensure that there is no inferred information about those users [45,46].

### Limitations

This study has a few limitations. First, we did not investigate the link between the mental health status of people with GAD and demographic characteristics, including age, gender, education, and employment status. These features cannot be retrieved from a user’s profile using the Twitter API.

One limitation is that this study did not cover a user’s medical and childhood history. Traumatic events in the early years of someone’s life can negatively impact mental health throughout their life. Investigating a patient’s medical history enhances the mental health diagnosis because it reveals their medical problems and the complications they went through, which may develop into psychological disorders. These factors contribute to increasing the likelihood of experiencing mental health disorders such as GAD.

Another limitation is that this study did not consider the severity of GAD on a scale from mild to severe. The severity of GAD may affect how it interferes with several aspects of daily living, so the degree to which GAD causes impairments may vary based on its severity. This study is also limited by the lack of prospective data, which makes causal inference impossible; thus, only cross-sectional inferences can be drawn.

Regarding data collection, Twitter’s API imposes limitations that might impact the comprehensiveness of collected datasets. According to the limit imposed by the Twitter API, only the 3200 most recent tweets can be retrieved. Another limitation in terms of the representativeness of the sample is that identifying potential members of a control group can pose a challenge as it might include individuals who experience GAD but choose not to disclose their diagnosis publicly. However, this study used a regular expression to search for self-reported diagnosis statements of a mental health disorder, which is a common technique used to identify individuals diagnosed with a mental health disorder on social media [24,40].

As future work, we intend to extend this study to conduct sentiment analysis of tweets, which could clarify any cues for GAD. In addition, a larger study could be conducted in which the data obtained from social media platforms could be combined with other information, such as medical history, gender, age, and employment status, to yield a more accurate picture of an individual’s mental health and contribute to developing a robust GAD detection model via social media. Moreover, using more advanced NLP techniques beyond regular expressions to identify individuals with GAD could significantly enhance the ability to identify people with GAD via their social media posts. For instance, using machine learning models trained on a broader set of linguistic features allows for identifying markers of GAD effectively.

### Conclusions

Our study provides several valuable insights into identifying the characteristics of GAD in social media networks. First, examining tweets from a linguistic perspective indicated that the language used by people with GAD reveals their mental health status. Second, thematic analysis of tweets highlighted which topics people with GAD tend to discuss more with others. Third, studying the behavior patterns showed how users with GAD interact with others, which provides more understanding of GAD.

In this study, we identified and characterized the digital markers associated with GAD by conducting a comprehensive analysis of social media activity from individuals diagnosed with GAD. Using content-based analysis techniques such as LIWC for linguistic analysis and GuidedLDA for thematic analysis, we thoroughly examined the language and themes in these individuals’ social media posts.

Furthermore, we determined significant behavioral differences by analyzing user- and tweet-level features, such as hashtag participation and interaction patterns. We also investigated the correlation between the themes identified by GuidedLDA and various metadata for both the GAD and control groups within our dataset. Our findings include 47 distinct GAD digital markers, with results presented in terms of means, SDs, *P* values, and Cohen *d*, highlighting these markers’ statistical significance and effect size.

In conclusion, understanding the behavior and tweet content of users diagnosed with GAD could help develop an assistant tool for diagnosis and recovery progress assessment to be used by mental health professionals. This assistant-diagnostic tool would integrate insights from patients’ social media content and behaviors into personalized treatment plans. This approach may help clinicians develop timely updated personalized treatment plans by delivering continuous support and timely interventions, enhancing communication with health care providers, encouraging engagement in activities, and customizing coping techniques.

## Appendix

Multimedia Appendix 1 Examples of paraphrased tweets for each theme.

## Abbreviations

API: application programming interface
DSM-5: Diagnostic and Statistical Manual of Mental Disorders, Fifth Edition
GAD: generalized anxiety disorder
LDA: latent Dirichlet allocation
LIWC: Linguistic Inquiry and Word Count
NLP: natural language processing
NLTK: Natural Language Toolkit
POS: part-of-speech

## Data Availability

The datasets generated or analyzed during this study are not publicly available due to adhering to Twitter’s policies regarding the distribution of data collected using its application programming interface, but they are available from the corresponding author on reasonable request.
