# Supplementary material for: Profiling Generalized Anxiety Disorder on Social Networks: Content and Behavior Analysis
Source: J Med Internet Res. 2025 Mar 20;27:e53399. doi: 10.2196/53399 (PMC11969129; doi:10.2196/53399)
Supplement: Multimedia Appendix 1 [file jmir_v27i1e53399_app1.docx]

Multimedia Appendix 1. Examples of paraphrased tweets for each theme.

| Theme | Example |
| --- | --- |
| Symptoms | “My anxiety levels have reached an all-time high.”  “I tend to overthink things.”  “I'm so exhausted. I cannot sleep because the worry I have been experiencing keeps me up.”  “Because I am always worried and anxious, I have recently developed several conditions.” |
| Life Problems | “I'm becoming agitated and anxious about the tasks at work that I have to complete.”  “I can't study well for my exam.” |
| Relationships | “I came this close to breaking up my relationship.”  “ I got divorced.” |
| Feelings | “I feel helpless.”  “I have lost interest in what I enjoy due to my mental health.” |
